# Influence of life history strategies on sensitivity, population growth and response to climate for sympatric alpine birds

**DOI:** 10.1186/1472-6785-12-9

**Published:** 2012-06-29

**Authors:** Kathy Martin

**Affiliations:** 1 Department of Forest Sciences, University of British Columbia, 2424 Main Mall, Vancouver, British Columbia V6T 1Z4, Canada; 2 Environment Canada, 5421 Robertson Road, Delta, British Columbia, V4K 3 N2, Canada; 3 Present address: Canadian Wildlife Service, Environment Canada, 115 Perimeter Road, Saskatoon, SK, S7N 0X4, Canada

**Keywords:** Alpine, Climate change, Elasticity, Life history variation, Population growth, Ptarmigan, Reproductive effort, Survival

## Abstract

**Background:**

The life history strategy of a species can influence how populations of that species respond to environmental variation. In this study, we used a matrix modeling approach to examine how life history differences among sympatric rock and white-tailed ptarmigan affect the influence of demographic rates on population growth (λ) and the potential response to a changing climate. Rock ptarmigan have a slower life history strategy than white-tailed ptarmigan in the study region with lower annual reproductive effort but higher adult survival.

**Results:**

Based on data from a 5-year field study, deterministic estimates of λ indicated that populations were stable for rock ptarmigan (λ = 1.01), but declining for white-tailed ptarmigan (λ = 0.96). The demographic rates with the highest elasticity for rock ptarmigan were the survival of after-second year females, followed by juvenile survival and success of the first nest. For white-tailed ptarmigan, juvenile survival had the highest elasticity followed by success of the first nest and survival of second-year females. Incorporating stochasticity into the demographic rates led to a 2 and 4% drop in λ for rock and white-tailed ptarmigan respectively. Using data from the first three years we also found that population growth rates of both species were depressed following an increased frequency of severe years, but less so for rock ptarmigan which showed greater resilience under these conditions.

**Conclusions:**

Our results provide evidence that populations of closely related species can vary in their response to environmental change as a consequence of life history differences. Rock ptarmigan, with a slower life history, are more responsive to demographic rates that influence survival and older life stages but this response is tempered by the extent of variability in each of the rates. Thus, predictions need to consider both aspects in modeling population response to a varying climate. Juvenile survival was a highly influential rate for both species, but the period from independence to first breeding is a poorly understood stage for many bird species. Additional study on juvenile survival, the influence of density dependence and the effects of predators as the mechanism driving survival-reproduction tradeoffs are all areas requiring further study.

## Background

Climate change is expected to result in higher mean temperatures and greater annual variability in weather for many regions of North America [1]. Different species will almost certainly vary in their response to these changes and one feature that may contribute to this variation is the species life history strategy. The contribution of the demographic rates to population growth depends on where a species lies along a life history continuum. Populations of short-lived species that mature early and have high annual reproductive effort tend to be most sensitive to variation in the reproductive rates and juvenile survival, while those of longer lived species that mature later and have lower annual reproductive effort, tend to be more sensitive to variation in adult survival [2-5]. Thus, an understanding of how climate affects demography and the sensitivity of a population to demographic change, should aid predictions on the response of populations to a changing climate.

Population matrix models are commonly used to estimate the sensitivity of a population to variation in the demographic rates and can include complex relationships between the environment and demography [6,7]. For example, Reed et al. [8] used this approach to show that deer mice (*Peromyscus maniculatus*) populations in Kansas could not persist if mean precipitation declined by more than 11%. Relationships between demography and inter-annual variability in weather can also be incorporated into models. Greater variability among years raises the likelihood that for any given year, the population may be reduced either to absolute extinction, or to some threshold size below which other factors such as demographic stochasticity or Allee effects prevent a recovery [9,10]. Saltz et al. [11] used matrix models to show how a predicted increase in the variability of precipitation might lead to greater fluctuations in population size and a higher extinction risk for Asiatic wild ass (*Equus hemionus*) in Israel (see also Hilderbrand et al. [12]).

We used demographic data from a 5-year population study (2004–2008) on sympatric rock and white-tailed ptarmigan in the southern Yukon Territory to develop a matrix population model and investigate how life history variation affects sensitivity, population growth and susceptibility to climate variation. These two species are specialists of tundra habitat and we previously showed that they differ in their life history strategies [13]. White-tailed ptarmigan invest more heavily in reproductive effort and have greater annual productivity than rock ptarmigan, but they also have lower annual adult survival and an age structure biased towards younger females. We tested how these differences influenced sensitivity of population growth to changes in the demographic rates and based on theory predicted that white-tailed ptarmigan would be more sensitive to variation in the reproductive rates, while rock ptarmigan would respond more strongly to variation in survival, particularly of the older age classes. Following this, we asked how each species might respond to a changing climate and examined this in two ways. We first looked at how a directional change in early spring (April-May) temperature might affect these populations. Canadian Regional Climate Models predict a mean increase in spring temperature of 1.4°C over the next four decades [14]. Warmer spring temperatures and earlier snowmelt lead to earlier breeding for both species, which subsequently affects productivity through greater opportunities for re-nesting [13]. We tested how this change in spring temperature might affect population growth rates and predicted that white-tailed ptarmigan would show a greater response if they are more sensitive to changes in the reproductive rates. Our second test focused on how greater environmental stochasticity might affect each species. The first three years of our study included two years that were warmer than average (2004–2005), followed by a very cold year during which the populations crashed, likely due to a combination of severe weather coupled with high avian predation on adults [15]. For this component, we used the demographic rates during these years to simulate the response and recovery of each species to differing frequencies of severe years.

## Results

### Deterministic model results

Over the 5-year study, asymptotic population growth rate (lambda, λ) of rock ptarmigan was approximately stable at λ = 1.013, while for white-tailed ptarmigan it was lower at λ = 0.957 (Table 1). The stable age distribution indicated that ASY (after-second year) females were the dominant age class for rock ptarmigan (61%), while white-tailed ptarmigan had a lower proportion of ASY females (45%). This age structure was similar to that observed over the course of the study where ASY females comprised 64% of known breeders for rock ptarmigan and 48% for white-tailed ptarmigan. The net reproductive rate of a female rock ptarmigan was 1.036 and thus approximately at replacement levels (Ro = 1), while rates for white-tailed ptarmigan were below this level at 0.919. Generation times were about 0.7 years longer for rock ptarmigan than white-tailed ptarmigan (Table 1).

**Table 1:** Asymptotic matrix properties for rock and white-tailed ptarmigan (values are the point estimates based on a deterministic model)

**Matrix property**	**Rock ptarmigan**	**White-tailed ptarmigan**
Asymptotic population growth rate (λ)	1.013	0.957
Stable age distribution (**w**):	0.39/0.61	0.55/0.45
(second year/after second year)		
Weighted reproductive value (**v**):	1.00/1.09	1.00/1.14
(second year/after second year)		
Net reproductive rate (Ro)	1.036	0.919
Generation time (T)	2.61	1.90

**Figure 1:**
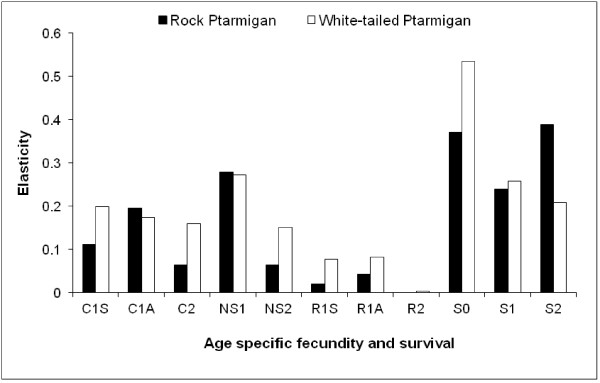
**Elasticity of survival and fecundity parameters for rock and white-tailed ptarmigan.** Rates include first clutch sizes for SY (C1S) and ASY (C1A) females, 2^nd^ clutch size (C2), nest success of first (NS1) and second attempts (NS2), first re-nest probabilities for SY (R1S) and ASY (R1A) females, second re-nest probabilities for ASY females (R2, white-tailed ptarmigan only), juvenile survival (S0), and, second year (S1) and after-second year (S2) survival.

To examine elasticity in greater detail, we decomposed the hatched young term into the underlying components of reproductive success. Predicted population growth rates for both species were very similar to earlier estimates using hatched young in the fecundity term (rock ptarmigan: λ = 1.002, white-tailed ptarmigan: λ = 0.961). For white-tailed ptarmigan, λ was most sensitive to changes in juvenile survival followed by nest success of the first attempt, survival of the SY (second year) age class and clutch size (Figure 1). For rock ptarmigan, λ was most sensitive to adult survival of the ASY age class, followed by juvenile survival, nest success of the first attempt and adult survival of the SY age class. In general, rates from the SY age class were more influential for white-tailed ptarmigan while those from the ASY age class were more influential for rock ptarmigan.

### Stochastic model results

Predicted population growth rates with a stochastic model were approximately 2 and 4% lower than deterministic predictions for rock and white-tailed ptarmigan respectively (Table 2). Population growth rates also varied depending on juvenile survival (S_0_) and the correlation between juvenile and adult survival. For rock and white-tailed ptarmigan, respectively, λ decreased by about 0.135 and 0.180 as mean juvenile survival rates declined from 0.32 to 0.22. A positive correlation between juvenile and adult survival depressed λ equally for both species, while a negative correlation enhanced λ although to a greater extent for white-tailed ptarmigan.

**Table 2:** Deterministic and median stochastic growth rates of rock and white-tailed ptarmigan based on matrix model projections

		**Rock ptarmigan**		**White-tailed ptarmigan**	
**Juvenile survival**	**Correlation**	**Deterministic lambda**	**Stochastic lambda (sd)**	**Deterministic lambda**	**Stochastic lambda (sd)**
0.22	0.5		0.917 (0.046)		0.818 (0.056)
0.22	0	0.944	0.923 (0.039)	0.864	0.826 (0.053)
0.22	−0.5		0.928 (0.033)		0.836 (0.045)
0.27	0.5		0.985 (0.048)		0.902 (0.063)
0.27	0	1.013	0.993 (0.042)	0.957	0.910 (0.056)
0.27	−0.5		0.999 (0.033)		0.923 (0.050)
0.32	0.5		1.052 (0.048)		0.997 (0.065)
0.32	0	1.082	1.062 (0.044)	1.048	1.000 (0.061)
0.32	−0.5		1.065 (0.038)		1.008 (0.057)

Models were run with three values for mean juvenile survival and three levels of within year correlation between mean juvenile and mean second-year/after-second year survival. Within year fertility and annual survival rates of yearlings and adults were assigned a correlation of 0.9. All other pairwise comparisons were assumed to have no correlation. Standard deviation for stochastic lambda estimates refer to the variation among 25-year projections.

### Effects of change in breeding season climate on population growth rates

The average date of first egg (DFE) for rock and white-tailed ptarmigan across the four years was 27 May and 30 May respectively. The expected average DFE assuming a 1.4°C rise in temperature was 23 May for rock ptarmigan and 24 May for white-tailed ptarmigan. This shift would be less than the variation observed over the four study years where average April-May temperatures varied over a range of 4.91°C and mean annual DFE varied from 21 May to 2 June for rock ptarmigan and 19 May to 7 June for white-tailed ptarmigan. A 1.4°C rise in mean spring temperature led to an expected 14% increase in the mean number of female hatched young per breeding female for rock ptarmigan and a 10% increase for white-tailed ptarmigan under the assumption that all other components of the ecosystem remain the same (see discussion for further detail). The enhanced reproductive output in turn increased λ for both species by about 0.05. Increasing the annual variability in the number of hatched young had only a minor influence on population growth rates of both species.

### Influence of severe years

**Figure 2:**
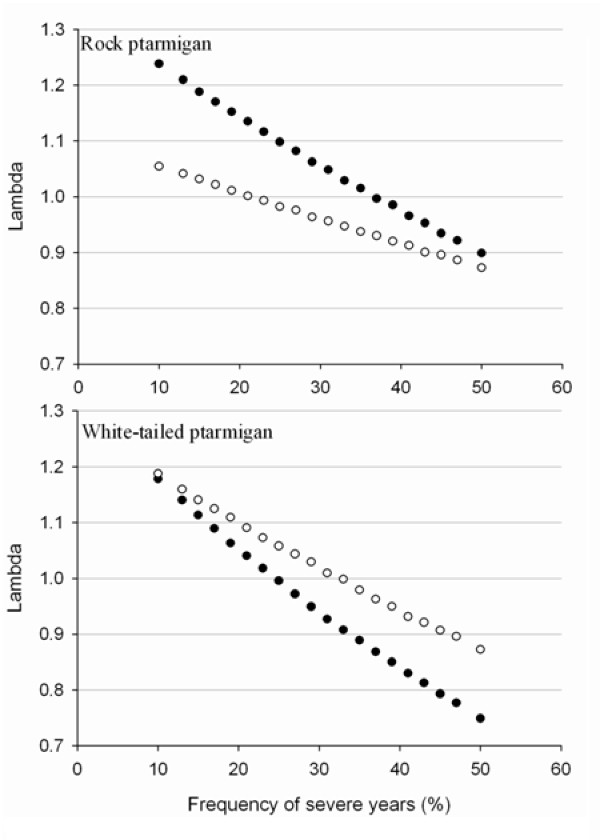
**Predicted lambda of rock and white-tailed ptarmigan populations in relation to the frequency of severe years**. Models were run with three projection matrices representing rates from 2004–2006. Solid circles refer to calculations of juvenile survival as a proportion of adult survival. Open circles refer to juvenile survival assuming a constant rate of 0.27. Values for all parameters are shown in Table 3.

The persistence of both species in relation to the frequency of severe years depended on the manner in which juvenile survival was specified (Table 3 shows the range of parameter estimates for each year). When we used the average estimate of 0.27 in the matrix, severe years could be no more frequent than 0.21 for rock ptarmigan and 0.32 for white-tailed ptarmigan to maintain stable populations (λ ≥ 1, Figure 2). When juvenile survival was calculated in relation to observed annual differences and as a function of adult survival, estimates for rock ptarmigan (S_JUV_ = 0.35) were considerably higher than for white-tailed ptarmigan (S_JUV_ = 0.24). Under these conditions, rock ptarmigan had greater resilience and could maintain a stable population even if severe years were as frequent as 0.36, while they could be no more frequent than 0.24 for a stable white-tailed ptarmigan population. Over the course of the study, only one of the four years had this level of severity (0.25 frequency), but without long-term data it is unclear how often these events occur.

**Table 3:** Mean parameter estimates for rock and white-tailed ptarmigan during two years of high productivity and survival (representing 2004–05), and one severe year of low productivity and survival (representing 2006)

**Parameter**	**Rock ptarmigan**			**White-tailed ptarmigan**		
	**2004**	**2005**	**2006**	**2004**	**2005**	**2006**
SY hatched young (female)	1.34	1.21	0.90	3.25 ± 0.25	2.11 ± 0.59	1.18 ± 0.43
ASY hatched Young (female)	1.88 ± 0.44	1.67 ± 0.43	1.07 ± 0.34	3.33 ± 0.33	2.50 ± 0.59	1.50 ± 0.54
Juvenile survival (method 1)	0.40	0.48	0.18	0.41	0.17	0.14
Juvenile survival (method 2)	0.27 (all years)			0.27 (all years)		
Adult female survival	0.67 ± 0.10	0.74 ± 0.09	0.41 ± 0.09	0.58 ± 0.22	0.55 ± 0.17	0.24 ± 0.10

These parameters were used in matrix projection models to predict population persistence under varying frequency of the severe year (see Figure 2). Juvenile survival estimates include values from the literature and therefore do not have associated standard errors. Because there were few SY female rock ptarmigan, rates were based as a proportion (0.8) of the ASY estimate using the results of previous studies (see text).

## Discussion

### Influence of life history on population growth

This study allowed for a comparison of how two congeneric and sympatric species differ in the influence of the demographic rates on λ, given that at this location, rock ptarmigan have a slower life history than white-tailed ptarmigan [13]. We found that adult survival (combined SY and ASY survival) had the highest elasticity for rock ptarmigan, while juvenile survival and the reproductive rates were relatively more influential for white-tailed ptarmigan. Moreover, demographic rates of older females had a greater influence for rock ptarmigan, while those of younger females did for white-tailed ptarmigan. These results are in accordance with general theory on how life history affects λ for a range of taxa [2,4,5]. A previous study on willow (*Lagopus lagopus*) and white-tailed ptarmigan also found that juvenile survival and the reproductive rates were relatively more influential for populations that have greater investment in reproduction but lower adult survival [16]. Similar studies on mammals show that those species that mature early and have high reproductive rates are more sensitive to the fecundity rates [17] and that the importance of the reproductive parameters on λ declines as maturation is delayed [3]. Incorporating the importance of different demographic rates on λ has become an important tool in management plans for species of concern [17-19].

An important question is to what extent are these findings characteristic of rock and white-tailed ptarmigan populations generally. The reproduction-survival trade-off between these two species in the Yukon may be related to how each responds to the environmental conditions of this particular location and might vary elsewhere. White-tailed ptarmigan in the Yukon tend to have higher reproductive effort than those in Colorado, although there are only slight differences in annual survival between the two populations [20]. For northern bobwhite (*Colinus virginianus*), populations in the northern part of the range have lower adult survival but greater reproductive output than those in the south [21], see also [22]. These examples suggest that populations vary in their demographic rates and life history across the range of a species. However, exceptions exist and in a similar study to ours, Novoa et al. [23] showed that demographic rates and life history strategies of two alpine rock ptarmigan populations in the Alps and Pyrenees of Europe were very similar to one another as well as to our Yukon population. In all of these cases, female annual survival rates averaged 0.60 to 0.70, which is considerably higher than what is often observed for willow and white-tailed ptarmigan where survival rates of 0.35-0.50 are more typical [16,20]. Coupled with the high annual survival of rock ptarmigan is a relatively low investment in reproduction with smaller clutch sizes and low re-nesting effort following failure [13,23].

Juvenile survival is one of the most difficult demographic rates to measure in birds and yet our results indicate that it is a highly influential parameter for ptarmigan populations. A change in the mean estimate from 0.32 to 0.22 with no change in the variance strongly affected λ of both species, but especially for white-tailed ptarmigan. Other studies on ptarmigan and grouse have also found juvenile survival to be an important determinant of population growth rates and/or the number of birds observed at the start of the next breeding period [16,24,25]. Similar results have been noted for other short-lived birds (e.g. passerines, [5]). For ptarmigan generally, dispersal and external recruitment, which is predominantly by juveniles, is key to regional population persistence and the recovery of local populations following declines [26,27]. We know little about juvenile survival or the factors affecting it for these and most other bird species. Future studies could examine the timing of movements by juveniles, distances moved from natal to breeding populations, estimates of survival from independence to the following breeding season and the effects of juvenile recruitment on population stability for each species. With additional years of study on the vital rates, it would also be useful to estimate the strength of density dependence and how that affects population growth rate e.g. [28]. Density dependence can help prevent extinction risk through elevated vital rates at low density, but can also limit the ability of the population to increase if density dependence then acts to lower reproduction and/or survival [10].

### Response of populations to changing climatic conditions

Using the projection matrices combined with empirical data, we found that population growth rates of both species would respond similarly (λ increase of 0.05) to a 1.4°C rise in mean temperature by mid-century. This increase was slightly reduced following greater variability in spring conditions. We showed earlier that white-tailed ptarmigan populations were more sensitive to changes in the reproductive rates and thus predicted that their lambda should respond more strongly to a rise in spring temperature. However, rock ptarmigan have a stronger positive relationship between temperature and the number of hatched young [13] and although less sensitive to variation in the reproductive rates, this appears to balance out the response of λ to a rise in temperature. These simulations also showed that populations of the two species would be approximately stable over the long-term as long as severe events, which simultaneously depress reproduction and survival, occurred at a frequency of less than once every four years. During our study, a single severe year led to a population crash of both species, similar to what has been observed for other sympatric grouse elsewhere [29].

A key assumption in using matrix models in this manner is that the demographic rates are only responding to a change in temperature, while all other environmental influences are constant, but this may be unlikely [7]. Because nest predators are a key determinant of reproductive output, accurate predictions would require knowledge on how climate warming would influence behavior, abundance and composition of the predator community. Our approach in this scenario only tested the effects of a changing spring climate but it would also be useful to simultaneously test the consequences of a changing climate during the breeding and non-breeding seasons. Climate models predict that by mid-century, winter temperatures in the region will rise by about 2°C while precipitation will increase by 10-20%. The combined effect of these changes is expected to reduce winter snowpack by about 25% [1]. Other studies on grouse have found both positive and negative effects of snow-depth on adult survival [30,31] and thus, how rock and white-tailed ptarmigan would be affected by this change is uncertain. Model predictions also need to incorporate changes in the avian predator community as they are typically the dominant factor affecting ptarmigan survival throughout the year [23,32,33].

## Conclusions

Northern alpine environments are predicted to experience an increase in both the mean and variability in spring temperatures and precipitation with continued climate warming [1]. The response of species to these changes is relatively unknown but what our study showed is that closely related species in the same environment can have different life history strategies and that in turn can determine how populations respond to environmental conditions. As expected, the species with a faster life history was more sensitive to traits that are associated with early life stages. However, this relationship was also influenced by the degree of variability in each trait in response to spring climate, an issue that is important for future predictions [7,10]. Although our simulations provide insight into how changes during the breeding season might affect these species, our certainty on these predictions requires more information on juvenile survival and the role of the non-breeding season on population growth. As noted, avian predators are often the dominant cause of mortality elsewhere and were in our study during the short breeding period. It is possible that this relationship with avian predators is one factor affecting the reproduction-survival tradeoff observed for these species and therefore, further study is needed on when the principal mortality occurs and how this could be affected by a shifting climate.

## Methods

### Study area and species

Field work was conducted from May through July of 2004–2007 on a 10 km^2^ alpine study site (Pika Camp) in the Ruby Range Mountains of the southern Yukon Territory, Canada. In 2008, we also returned to the site to census marked individuals to allow 4 years of survival data. Mean daily temperature at the site averaged 6.5°C during the breeding period (May through July) and −13.3°C during the winter months (November through March).

Rock and white-tailed ptarmigan are precocial, ground-nesting birds within the genus *Lagopus* (family Phasianidae). Ptarmigan are socially monogamous during breeding although polygyny occurs regularly. Males of these two species remain with the female on the territory until approximately mid-incubation after which they leave and form small flocks with other males and failed female breeders. Females will re-nest following clutch failure but only produce one brood per year, which stay with the female until late August to late September [13,15,16,27]. The two species are sympatric in the study region but show some segregation in their breeding habitat and display intra- and inter-specific territoriality. Rock ptarmigan typically select lower alpine meadows while white-tailed ptarmigan select steeper, rocky slopes at higher elevations [34]. Densities of rock and white-tailed ptarmigan respectively ranged from about 4–5 and 2–3 pairs/km^2^. For additional detail on habitat and other study site characteristics see Wilson and Martin [13,34].

### Field methods

We used ground nets and noose poles to capture males and females during the period after territory establishment and prior to nesting (~ 1 May – 20 May). All individuals were color-marked with an aluminum band on one leg and a numbered plastic color band on the other. Females were fitted with a 4 or 7 g radio-transmitter (Holohil Inc., Carp, Ontario) to facilitate nest finding. Individuals were aged as second-year (SY, 10–11 months) or after-second year (ASY, 22+ months of age) based on the pigmentation on the outer primaries and primary coverts following Weeden and Watson [35]. The total number of breeding females monitored for reproduction by age class were as follows: rock ptarmigan SY - 11, rock ptarmigan ASY - 65, white-tailed ptarmigan SY - 23, white-tailed ptarmigan ASY – 28 (note that some individuals were monitored as both SY and ASY birds as well as multiple years as ASY birds). Date of first egg was estimated by observing nests during laying, back-dating from hatch or floating an egg during incubation following the method of Westerskov [36]. Incubation was assumed to have begun with the laying of the penultimate egg [37,38]. Clutch size for first and second attempts was determined as the maximum number of eggs laid per nesting attempt (i.e. completed clutch), defined as those where egg number was constant over two consecutive nest checks. In the lining of each nest we also placed a small Ibutton temperature logger (Maxim Products, Dallas, TX), which keeps a continuous record of nest temperature and allowed us to determine the precise time of nest failure or hatch. During incubation, nests were checked visually every 3–5 days to determine if they were still active and females were only flushed off the nest when we needed to switch the Ibutton every 10–12 days. We monitored nests more frequently as the expected hatch date approached to ensure an accurate measurement of the number of chicks hatched and when they left the nest. Broods were re-located every 3 to 7 days to re-count the chicks to estimate juvenile survival. If a nest failed, the female was located every few days to determine whether and when she initiated a re-nest attempt.

Radio-collars were removed from two-thirds of the females after breeding. For the other one-third, we left the collars on through the winter and this allowed us to conduct surveys of the immediate study area and adjacent regions (approximately 100 km^2^) in the following spring to estimate the extent of breeding dispersal. With these surveys, we found no evidence that females of either species that had bred at the study site in the previous year had moved elsewhere within this 100 km^2^ range. We cannot rule out the possibility that they had moved beyond this range but overall this suggests that our estimates of apparent survival are likely close to true survival and not biased by permanent emigration from the study area. The use of animals in this research adheres to the ethical standards of Canada as approved by the University of British Columbia Animal Care Committee (permit no. A05-0450).

### Calculation of demographic rates

Many demographic rates used here were previously estimated in Wilson and Martin [13,20], but we summarize those analyses used in our population model. Because of the ASY-biased age structure for rock ptarmigan, we had a low sample size of SY females to estimate the mean and variance in the number of hatched young they produced. Data for white-tailed ptarmigan indicated that SY females hatched about 20% fewer young than ASY females and this difference is similar to average estimates for both species elsewhere [16,39,40]. Therefore, we estimated the mean and variance in the number of hatched young for ASY female rock ptarmigan and assumed this rate would be 20% lower for SY females with equal variance. Among ptarmigan generally, the probability of re-nesting is age dependent with 2 and 3 year-old females showing a greater propensity to re-nest than 1-yr old females [41,42]. We calculated the observed re-nest probability for all individuals combined and adjusted by age assuming SY females had a 20% lower re-nest propensity than ASY females. We previously found no influence of female age on daily nest survival [15]. Therefore we assumed constant nest survival with age for both species and estimated rates separately for first and second nest attempts.

We used program MARK for analyses of adult survival [43]. Adult survival was previously calculated in Wilson and Martin [13] but we expanded on previous estimates to obtain separate survival rates for SY and ASY females of each species. Juvenile survival was directly estimated during the chick stages (June through August) and both species had similar rates with a mean of 0.52-0.55 over this period [15,20]. To determine annual juvenile survival, we combined these values with an estimate from September through April from the literature. Survival of juvenile willow and white-tailed ptarmigan from September to the following spring averages about 0.45-0.55 [44]. There are no detailed studies of juvenile survival in rock ptarmigan. To parameterize the population model, we assumed a rate of 0.5 from independence through April, which when combined with our field data prior to independence yield an average annual rate of about 0.27. However, because of uncertainty in this estimate, we ran most model projections with mean values of juvenile survival ranging between 0.22 and 0.32.

### Population model

Population growth rates were calculated using female-based matrix models [6,7,10] calculated in Matlab Vers 7.1 [45]. Variation in the size and age-structure of a population from time *t* to time *t* + 1 can be computed from:

(1) $$n_{\text{t}+1}=An_{\text{t}}$$

where n is a vector describing the age, stage or size-structure of the population and A is a population projection matrix. We used a two-age pre-breeding model with second-year (SY) and after-second year (ASY) females as the two classes:$A=\left[\begin{array}{cc} HY_{\mathrm{SY}}*S_{\mathrm{JUV}} & HY_{\mathrm{ASY}}*S_{\mathrm{JUV}}\\ S_{\mathrm{SY}} & S_{\mathrm{ASY}} \end{array}\right]$where HY_SY_ and HY_ASY_ are the number of female young hatched annually by SY and ASY females, S_JUV_ is the survival rate of juveniles from hatch to the following breeding season, and S_SY_ and S_ASY_ are the survival rates of adult SY and ASY females, respectively.

From the above model we calculated the population growth rate λ_1_ (the dominant eigenvalue) and from λ_1_, the subdominant eigenvalue (λ_2_), reproductive value (v, left eigenvector) and the stable-age distribution (w, right eigenvector). We also determined the average number of female offspring produced per female over her lifetime (net reproductive rate, Ro) as:$\text{Ro }=\sum_{x=0}^{n}{\text{s}}_{\text{x}}\;{\text{ f}}_{\text{x}}$where s_x_ is the probability of survival to age x and f_x_ is the fecundity of females at age x. After determining the net reproductive rate for both species, we calculated the generation time as:

(2) $$\text{T}=\text{ln}\left(\text{Ro}\right)/\text{ ln}\left(\lambda_{1}\right)$$

Matrix models are also useful for calculating elasticity [6,7,10], the proportional change in λ_1_ in response to a proportional change in a demographic rate r_i_.

(3) $${\text{Er}}_{\text{i}}=\partial \lambda_{1}/\lambda_{1}/\partial {\text{r}}_{\text{i}}/{\text{r}}_{\text{i}}$$

Although elasticity measures provide an estimate of how influential a particular demographic rate is, it is important to note that the variability in a rate must also be considered to predict which rates ultimately have the greater influence on population growth. To provide a more informative analysis of elasticity, we decomposed the fecundity terms (HY_SY_ and HY_ASY_) into the primary rates that influence the number of young hatched; clutch size, nest success and the probability of re-nesting after failure. This model had the following structure:$A=\left[\begin{array}{cc} F_{\mathrm{SY}} & F_{\mathrm{ASY}}\\ S_{\mathrm{SY}} & S_{\mathrm{ASY}} \end{array}\right]$For rock ptarmigan, F1 and F2 are equal to:

(4) $${\text{F}}_{\text{SY}}=\left[{\text{C}1}_{\text{SY}}\;\text{ns}1+\left(1-\text{ns}1\right)\;{\text{r}1}_{\text{SY}}\;\text{C}2\;\text{ns}2\right]\;{\text{ S}}_{\text{JUV}}\;0.5$$

(5) $${\text{F}}_{\text{ASY}}=\left[{\text{C}1}_{\text{ASY}}\;\text{ns}1+\left(1-\text{ns}1\right)\;{\text{r}1}_{\text{ASY}}\;\text{C}2\;\text{ns}2\right]\;{\text{ S}}_{\text{JUV}}\;0.5$$

where C1_SY_ and C1_ASY_ are the size of the first clutch for SY and ASY females respectively, C2 is the size of the 2nd clutch (equal for both age groups), ns1 and ns2 is nest success for the first and second attempt respectively, r1_SY_ and r1_ASY_ are the re-nest probabilities for SY and ASY females, and S_JUV_ is juvenile survival. Fifty percent of the young were assumed to be female and therefore estimates were multiplied by 0.5. Equations for white-tailed ptarmigan are the same except ASY females were assumed to have a probability of re-nesting a second time [13]:

(6) $${\text{F}}_{\text{SY}}=\left[{\text{C}1}_{\text{SY}}\;\text{ns}1+\left(1-\text{ns}1\right)\;{\text{r}1}_{\text{SY}}\;\text{C}2\;\text{ns}2\right]\;{\text{ S}}_{\text{JUV}}\;0.5$$

(7) $${\text{F}}_{\text{ASY}}=\left[{\text{C}1}_{\text{ASY}}\;\text{ns}1+\left(1-\text{ns}1\right)\;{\text{r}1}_{\text{ASY}}\;\text{C}2\;\text{ns}2+\left(1-\text{ns}1\right)\left(1-\text{ns}2\right)\;{\text{ r}1}_{\text{ASY}}\;{\text{ r}2}_{\text{ASY}}\;\text{C}2\;\text{ns}2\right]\;{\text{ S}}_{\text{JUV}}\;0.5$$

where r1_ASY_ and r2_ASY_ are the ASY re-nest probabilities for 2nd and 3rd attempts. If a 3rd attempt was initiated, we assumed clutch size and nest success was the same as for the second attempt. For both species, survival of SY and ASY females were represented by S1 and S2 as for the previous matrix.

The above estimates were calculated from a deterministic model without variation. To better examine the potential for uncertainty we also examined growth rates using a stochastic population model [46]. We introduced environmental stochasticity to the model by allowing demographic rates to be drawn at random from a specified distribution and simulated 1000 population trajectories each for 25 years following the approach of Morris and Doak [10]. The number of hatched young were randomly drawn from a stretched beta distribution with mean and variance equal to the observed values across the four years. The maximum and minimum values for this distribution were assigned based on likely upper and lower limits for the two species [37,38]. Annual survival of second year and after-second year females were assumed equal and drawn from a beta distribution with mean and variance approximated from field data. For earlier analyses we separated process and sampling variance in survival using the approaches outlined in [10]. However, the resulting estimates of process variance were low relative to sampling variance and led to a range of annual survival rates that appeared to be too narrow given knowledge of species biology. Therefore, we chose to use total variance in our models instead even though it is a conservative approach that includes process and sampling variance e.g. [41,47]. Annual juvenile survival was also drawn from a beta distribution but simulations were run with a mean rate of 0.22, 0.27 and 0.32 to incorporate the uncertainty described earlier.

To include the potential effects of covariation in the vital rates on population performance [46,48], we allowed rates to be correlated within years. Because only 3 and 4 years of data were available for survival and fecundity respectively, it was not possible to examine correlations among demographic rates and therefore, we assigned correlations based on likely values given the species biology. Within-year correlations between SY and ASY fecundity and, between SY and ASY survival are likely strong and we assigned a correlation coefficient (r) of each = 0.9. Annual survival rates of juveniles and adults (combined SY and ASY) may also be correlated although the relationship could vary. A negative relationship might be observed if there are strong density-dependent effects of adults on juveniles, while a positive relationship may be more likely if environmental conditions affect all age groups equally. To represent this range of possibilities, we ran the model with juvenile-adult survival correlations = −0.5, 0 and 0.5, and evaluated how each affected population growth. We assumed there were no within-year correlations between the reproductive and survival components. Between-year correlation might occur when factors such as climatic conditions, predator abundance, disease or food supply are temporally autocorrelated [10,49]. However, because of the difficulty in identifying these effects with short term data, we assumed there were no between-year correlations in this analysis. Density dependence is also a key process in population dynamics but is difficult to incorporate in matrix projection models because many years of data are required to identify the functional relationship [6,10]. Rather than assume what this relationship might be, we chose not to include density dependence here.

### Effects of climate variation during the breeding season on population dynamics

We also investigated the effects of a change in breeding season climate on population growth rates of each species. We used the Canadian Regional Climate Model (CRCM) 3.6 produced by the Canadian Centre for Climate Modelling and Analysis [14] to create scenarios for future climate by 2050.

#### Scenario 1

We first estimated the influence of a change in mean spring (April-May) temperature on population growth. In Wilson and Martin [13] we showed that warmer temperatures lead to earlier breeding, larger clutches and a greater number of young hatched. The CRCM model was used to estimate change in mean spring temperatures in the study area from the present until 2050 and predicted a 1.4°C rise over that period [14]. To incorporate this effect in the first scenario, we used the previously determined relationships between timing of breeding and spring temperature to estimate the predicted change in breeding date relative to the average during the study. We then used the relationship between breeding date and number of hatched young to estimate how the number of hatched young might change over time. This estimate was incorporated in the projection matrix and we simulated 5000 runs of stochastic population growth each over 25 years. All parameters were drawn from the same distributions as noted earlier and for this analysis, we assumed there was no change in the variance.

#### Scenario 2

To examine how populations of each species respond to severe events such as that observed in 2006, we parameterized three projection matrices with values of hatched young and adult survival as observed in 2004, 2005 and 2006. Only these three years were used because the population crash in 2006–2007 resulted in smaller sample sizes in the spring of 2007. For this type of analysis, a particular matrix element always occurs with the other elements in that year and it may be more appropriate to assume that juvenile survival was higher than average in the two good years and lower in the severe year. Hannon and Martin [44] note that ptarmigan juvenile survival from independence to recruitment tends to be about 15-25% lower than adult survival. Therefore, to estimate juvenile survival for each of the three years, we used annual measures of chick survival through August and assumed survival from September through April to be 20% lower than adult survival. Because of the uncertainty in this parameter, we also ran a separate set of analyses using the original average of 0.27 for both species. We then ran model simulations where the frequency of the severe year varied between 10 and 50 percent. For each case, 1000 population trajectories were run for 25 years and we examined the population growth rate as for scenario 1.

## Competing interests

The authors declare that they have no competing interests.

## Authors' contributions

SW contributed to the development and the design of the study, conducted field work, performed analyses and drafted the manuscript. KM contributed to study design and development, interpretation of the results and aided with manuscript writing and editing. Both authors read and approved the final manuscript.

## Authors' information

This study was part of a doctoral dissertation by SW under the supervision of KM in the Department of Forest Sciences, University of British Columbia.
